# Monitoring Needs for Gene Drive Mosquito Projects: Lessons From Vector Control Field Trials and Invasive Species

**DOI:** 10.3389/fgene.2021.780327

**Published:** 2022-01-06

**Authors:** Gordana Rašić, Neil F. Lobo, Eileen H. Jeffrey Gutiérrez, Héctor M. Sánchez C., John M. Marshall

**Affiliations:** ^1^ Mosquito Genomics, QIMR Berghofer Medical Research Institute, Brisbane, QLD, Australia; ^2^ Department of Biological Sciences, University of Notre Dame, Notre Dame, IN, United States; ^3^ Divisions of Epidemiology and Biostatistics, School of Public Health, University of California, Berkeley, Berkeley, CA, United States; ^4^ Innovative Genomics Institute, University of California, Berkeley, Berkeley, CA, United States

**Keywords:** population replacement, population suppression, *Wolbachia*, RIDL, invasive species, resistant alleles, gene drive, monitoring

## Abstract

As gene drive mosquito projects advance from contained laboratory testing to semi-field testing and small-scale field trials, there is a need to assess monitoring requirements to: i) assist with the effective introduction of the gene drive system at field sites, and ii) detect unintended spread of gene drive mosquitoes beyond trial sites, or resistance mechanisms and non-functional effector genes that spread within trial and intervention sites. This is of particular importance for non-localized gene drive projects, as the potential scale of intervention means that monitoring is expected to be more costly than research, development and deployment. Regarding monitoring needs for population replacement systems, lessons may be learned from experiences with *Wolbachia*-infected mosquitoes, and for population suppression systems, from experiences with releases of genetically sterile male mosquitoes. For population suppression systems, assessing monitoring requirements for tracking population size and detecting rare resistant alleles are priorities, while for population replacement systems, allele frequencies must be tracked, and pressing concerns include detection of gene drive alleles with non-functional effector genes, and resistance of pathogens to functional effector genes. For spread to unintended areas, open questions relate to the optimal density and placement of traps and frequency of sampling in order to detect gene drive alleles, drive-resistant alleles or non-functional effector genes while they can still be effectively managed. Invasive species management programs face similar questions, and lessons may be learned from these experiences. We explore these monitoring needs for gene drive mosquito projects progressing through the phases of pre-release, release and post-release.

## Introduction

As the impact of currently-available tools to control malaria stagnates, gene drive mosquitoes have been described as a promising and potentially transformative technology. Exciting progress has been made in *Anopheles* vector species towards two general classes of strategies: i) population replacement, whereby inheritance is biased in favor of an allele that confers refractoriness to pathogen transmission (Adolfi et al., 2020; Carballar-Lejarazú et al., 2020), and ii) population suppression, whereby vector populations are suppressed by biassing inheritance in favor of an allele that induces a severe fitness cost or sex bias (Hammond et al., 2016; Kyrou et al., 2018). A key strength and challenge facing this technology is its potential scale of impact, and as products advance from contained laboratory testing to semi-field testing and small-scale field trials, monitoring programs must be envisioned that align with the scale of intervention (James et al., 2018).

While *Anopheles* gene drive projects pose challenges, precedents exist from monitoring of biological and self-limiting genetic control trials, albeit in *Aedes* mosquitoes (Hoffmann et al., 2011; Carvalho et al., 2015; Utarini et al., 2021). Field trials of *Wolbachia*-infected *Aedes aegypti* in Queensland, Australia tracked the frequency of *Wolbachia* infection in the mosquito population over time, including heterogeneity in space and spread to areas neighboring the trial site (Hoffmann et al., 2011; Schmidt et al., 2017), and more recently, a randomized control trial in Yogyakarta, Indonesia, monitored dengue incidence in the human population (Utarini et al., 2021). Field trials of releases of *Ae. aegypti* carrying a dominant lethal gene (RIDL) intended for population suppression tracked mosquito population density over time with a high degree of spatial resolution (Carvalho et al., 2015). On a wider scale, precedents also exist for invasive species monitoring programs (Koch et al., 2020).

Here, we draw from these programs to explore the monitoring needs of *Anopheles* gene drive projects as they move through the phases of pre-release, release and post-release (Table 1). We consider distinct requirements for population replacement versus suppression, focusing on low-threshold approaches.

**TABLE 1:** Monitoring priorities for *Anopheles* gene drive projects as they move through the phases of pre-release, release and post-release. “R” refers to population replacement, and “S” refers to population suppression.

Question/endpoint	Activity	Indicators	Priority		
			Pre-release	During release	Post-release
Target mosquito vector abundance	Adult and pupal sampling throughout the study area and across seasons	Number of adults per trap per period, number of pupae per breeding site per period	High (R, S)	Medium (R), High (S)	Medium (R), High (S)
Environmental drivers of mosquito population	Measuring environmental data within and across seasons	Daily rainfall, temperature, humidity etc.	High (R, S)	High (R, S)	Medium (R, S)
Target mosquito local-scale movement	Mark-release-recapture experiments	Local dispersal kernel, average dispersal distance	High (R, S)	Medium (R, S)	Low (R, S)
Target mosquito intermediate and wide-scale movement	Adult and larval sampling over a wide scale and population genetic analysis	Effective dispersal distance, migration rates at larger spatial scales	High (R, S)	Medium (R, S)	Low (R, S)
Target mosquito insecticide resistance	Larval sampling, rearing and laboratory testing	Fraction of knockdown and dead mosquitoes after exposure to insecticide	High (R), Medium (S)	High (R), Low (S)	Medium (R), Low (S)
Target and non-target vector biting rates	Adult sampling by human landing catch, or proxy	Human biting rate (mosquitoes per person per night)	High (R, S)	Medium (R), High (S)	Medium (R), High (S)
Target and non-target vector competence	Larval sampling and laboratory rearing and testing	Fraction of exposed mosquitoes with disseminated infection	High (R, S)	High (R), Medium (S)	High (R), Medium (S)
Target and non-target vector sporozoite rate	Adult sampling, dissection and microscopy	Fraction of examined mosquitoes with sporozoites	High (R, S)	High (R), Medium (S)	High (R), Medium (S)
Malaria incidence and prevalence	Passive case detection, cohort studies, cross-sectional surveys	Health system case reports, cohort-based incidence, cross-sectional prevalence	High (R, S)	High (R, S)	High (R, S)
Prevalence of gene drive allele in target species	Adult and larval sampling in target and non-target areas and molecular assays	Allele frequency throughout the study area and allele presence elsewhere over time	N/A	High (R, S)	High (R, S)
Presence of gene drive allele in non-target mosquito species	Adult and larval sampling in target and non-target areas and molecular assays	Allele presence in non-target species	N/A	Medium (R, S)	Medium (R, S)
Phenotypic stability of gene drive construct in target mosquito species	Adult and larval sampling in target and non-target areas and laboratory testing	Gene drive efficacy, effectiveness of effector gene (R), sex bias or fitness cost (S)	N/A	High (R, S)	High (R, S)
Fitness of gene drive mosquitoes	Fitting models to data, parameter estimation	Male mating competitiveness, female fecundity, adult lifespan	N/A	High (R, S)	High (R, S)
Prevalence of resistance to gene drive in target mosquito species	Adult and larval sampling, molecular assays, laboratory evaluation	Prevalence of drive-resistance mechanisms throughout the study area	High (R, S)	Medium (R), High (S)	Medium (R), High (S)

## Pre-Release Monitoring

Pre-release monitoring data serves a range of purposes. First, it allows us to better understand the temporal dynamics of mosquito populations, their drivers, and effective sampling tools. Second, it enables us to design efficient release strategies that enhance the likelihood of intervention success, and to minimize risks such as escape of transgenic organisms to non-target populations. And third, baseline data provides an understanding of intervention-related changes both to the vector population and to vector-borne disease transmission.

One of the most common forms of pre-release monitoring data for genetic and biological control systems is the temporal measure of local mosquito population size, alongside environmental data such as temperature and rainfall. Population size estimates and seasonal patterns help to inform the timing and size of releases for both suppression and replacement gene drive systems, and represent a fundamental entomological endpoint to assess the efficacy of a suppression strategy. Highly seasonal mosquito population dynamics, such as in the Sahel of Africa, are especially important to monitor, as gene drive modeling studies reveal a large influence of seasonality on the outcome of a control program (North et al., 2019).

Effective sampling tools to measure relative mosquito population density depend on the species and location of interest. For the RIDL trial in Juazeiro, Brazil, and the *Wolbachia* trial in Queensland, *Ae. aegypti* was the main species of interest and a combination of ovitraps and BG Sentinel traps were used, with human landing catches (HLCs) also being used for the RIDL trial (Hoffmann et al., 2011; Carvalho et al., 2015). For *Anopheles* species, evaluation of sampling devices that accurately represent local mosquito densities is needed, and selection of these tools will depend on local regulations (e.g., HLCs are not always permissible). Most entomological monitoring is directed at adult female mosquitoes, so details of the adult male population may need to be inferred from captures of adult females and juvenile stages.

Monitoring data on mosquito movement patterns, although difficult to collect, is essential prior to field trials of gene drive mosquitoes. This can help to inform: i) the spatial resolution required for releases, and ii) the risk of escape to non-target populations. Data on autonomous dispersal of *Ae. aegypti* has been used to determine the size of buffer zones around treatment areas for suppression programs, and the size of the release area for the *Wolbachia* replacement programs (Schmidt et al., 2017). Similar *Anopheles* data could inform gene drive programs. Mark-release-recapture experiments are an effective way to estimate *Anopheles* dispersal patterns on a village or suburban scale (Taylor et al., 2001; Epopa et al., 2017), and have previously been used to quantify movement patterns of genetically sterile male *Aedes* prior to field trials (Lacroix et al., 2012). Population genetics offers complementary tools to infer local (Filipović et al., 2020) and intermediate to large-scale movement patterns that result from human-assisted dispersal (Marsden et al., 2013). This can help to identify potential routes of escape, with monitoring encompassing mosquito populations close to the target population or those connected via transport routes.

One form of pre-release monitoring uniquely required by gene drive mosquito projects is assessment of DNA sequence polymorphisms at the specific genetic locus targeted by the drive system. Some of these alternate alleles may confer a drive-resistant phenotype to the mosquitoes carrying them. For population suppression strategies, these confer a significant selective advantage over intact drive alleles, preventing the success of suppression programs. For population replacement strategies, alternate alleles may have a mild selective advantage over intact drive alleles; but even if not, could prevent the drive system from reaching a high frequency in the population. Modeling studies suggest that pre-existing drive-resistant alleles with population frequencies less than ~1% are tolerable for population replacement programs (Lanzaro et al., 2021). A recent study found an abundance of conserved sites that could potentially be targeted by gene drive systems by screening *Anopheles* specimens from the UC Davis Vector Genetics Lab archive and *An. gambiae* 1,000 Genomes Consortium (Schmidt et al., 2020). Modeling would ideally inform pre-release monitoring requirements, with samples corresponding to the location or region of interest.

Pre-release monitoring should focus on the target species, but include some consideration of: i) other local vector species for the pathogen of interest, ii) species between which there is some gene flow, albeit at potentially low levels, and iii) species that may compete for a similar niche (for population suppression strategies). Other local vector species are important to quantify in order to understand the proportion of pathogen transmission attributed to the target species, and hence the expected impact on disease transmission. Species between which there is a low level of gene flow could result in spread of the construct with ecological consequences. For *Anopheles*, limited gene flow occurs between members of the *An. gambiae* species complex, which includes *An. coluzzii*, *An. arabiensis*, and others (Taylor et al., 2001; Weetman et al., 2014). The potential for niche replacement is an important consideration for population suppression strategies where non-target species have some vectorial capacity.

## Monitoring During a Release

Monitoring during a release serves three key purposes. First, it allows us to monitor the progress of the release, as measured by changes in genotype frequencies and population size. This also allows us to adapt the release scheme in an iterative fashion. Second, it allows us to compare changes in the vector population and, ideally, vector-borne disease incidence, to the pre-release baseline or control area. And third, it allows us to assess biosafety features such as confinement to a trial site.

For population replacement systems, there is a need to monitor: i) how intact drive alleles spread through the population, ii) the extent to which drive-resistant alleles emerge and spread, and iii) effectiveness of the effector gene. As a case study in *Aedes*, initial trials of *Wolbachia*-infected *Ae. aegypti* in Queensland demonstrated how a network of ovitraps (∼1 per two houses) and BG Sentinel traps (∼1 per 30–45 houses) combined with a PCR assay to determine mosquito species and *Wolbachia* status successfully documented the spread of *Wolbachia* over time (Hoffmann et al., 2011). For population replacement strategies in *Anopheles*, additional assays will be needed to monitor for the intact drive allele and alternative alleles in target mosquito species, as well as their presence in non-target species, which can be efficiently achieved through targeted NGS amplicon sequencing, for instance.

For population suppression systems, there may be no effector gene to monitor, but there is a need to monitor the stability of suppression phenotype (e.g., fecundity reduction, lethality of juvenile stages, or sex ratio bias) and reduction in mosquito density. As a case study in *Aedes*, a grid of ovitraps spanning treated and control areas was used to provide an indirect measure of adult *Ae. aegypti* abundance for the RIDL trial in Juazeiro (Carvalho et al., 2015). Larvae were also scored for the transgene based on a red fluorescent marker phenotype, and non-fluorescent larvae were reared to adults to check for other vector species. For population suppression gene drive projects in *Anopheles*, it is especially important to assay for drive-resistant alleles which, due to their selective advantage over intact drive alleles, are expected to rapidly spread through populations following emergence. These assays can be informed, in part, by experiments on caged populations that mimic genotype fixation and can be designed to rapidly select and identify functional resistance alleles among the detected variants in CRISPR-based suppression gene drives (Fuchs et al., 2021). For large-scale interventions, it will be essential to detect these alleles quickly if their spatial spread is to be curtailed.

Close monitoring of intervention progress is highly valuable as it enables releases to occur in an adaptive and iterative manner. For instance, as *Wolbachia*-infected *Ae. aegypti* were released and monitored in Queensland, the fitness cost parameter estimate was refined and model predictions ensured the release scheme would result in *Wolbachia* exceeding its threshold frequency (Hoffmann et al., 2011). For releases of *An. gambiae* with low-threshold gene drive systems, ongoing monitoring will allow us to refine model parameters and ensure release schemes achieve entomological and epidemiological targets. Parameters such as mating competitiveness and adult lifespan are essential to refine, as lab measurements are not reliable in the field.

In seeking to understand the drivers of pathogen transmission when a gene drive intervention targets only one of several local vector species, multiple sampling tools will be needed to capture entomological indicators of transmission corresponding to each local vector species. Sampling tools should be selected that take advantage of vector species behaviors, and that collect data reflective of the question to be addressed. Other factors should be monitored that could explain potential epidemiological outcomes, such as the inclusion of additional interventions as part of the gene drive release (e.g., insecticide-treated nets), and the inclusion of insecticide-susceptibility in the released modified mosquitoes, potentially combined with ongoing spread of insecticide resistance alleles in the local wild mosquito population. Epidemiological data is often detected passively through reported cases of symptomatic disease, and should be compared leading up to, during and after the release (Utarini et al., 2021).

Lastly, monitoring during a release allows biosafety features of a gene drive intervention to be assessed - most importantly, confinement to the target area. As a case study, monitoring of *Wolbachia*-infected *Ae. aegypti* in Queensland included a site across a major highway from one release site, and another separated by over 1 km from the second release site (Hoffmann et al., 2011). *Wolbachia* was sporadically detected in both non-release locations, suggesting occasional movement of *Ae. aegypti* spanning more than 20 times their average dispersal distance (Muir and Kay, 1998; Russell et al., 2005). *Anopheles* mosquitoes disperse greater distances than *Aedes* (Guerra et al., 2014), suggesting the potential for low-threshold gene drive systems in *Anopheles* to spread on a wide-scale. This highlights the need for rigorous monitoring of non-target populations during trials and interventions, even more so for *Anopheles*, including at sites that could facilitate wide-scale spread, such as nearby sea and airports.

## Post-release Monitoring

Post-release monitoring is needed at multiple scales. At the scale of the release and its immediate vicinity, it is needed to monitor the continued persistence and effectiveness of the intervention, and on a wider scale, it is needed to assess the extent to which a low-threshold gene drive system has spread spatially, as well as the extent to which alternative alleles are spreading.

Local-scale monitoring needs depend on the gene drive system being implemented. For population replacement systems, it is important to monitor: i) persistence of intact drive alleles, ii) prevalence of drive-resistant alleles, and iii) continued effectiveness of the effector gene. A key concern for these systems is that drive-resistant alleles may emerge that are less costly than intact drive alleles, as when there are fewer cleavable wild-type alleles left in the population, these resistant alleles may replace the drive alleles (Eckhoff et al., 2017). Monitoring for this scenario will be essential in the aftermath of a successful release. Loss of effector gene function is also essential to monitor post-release, as this could conceivably happen in the months and years following a release, whether through loss-of-function mutations in the effector gene, evolution of effector gene-resistant pathogen strains, or some combination thereof (Marshall et al., 2019).

For population suppression systems, it is important to monitor: i) target species population size, ii) presence and abundance of non-target vector species, iii) presence of drive-resistant alleles, and iv) persistence of intact drive alleles. Modeling of population suppression drive systems in spatially-structured populations warns against the expectation of local target species elimination. Although this is a possibility for an ideally-functioning drive system, a more likely scenario is a fluctuating level of suppression, as populations are repeatedly eliminated and recolonized by wild and drive-carrying organisms (Eckhoff et al., 2017; North et al., 2019). This means that populations must continue to be monitored even following an initially successful release. In addition to monitoring the target species, local non-target vector species should be closely monitored, as suppression programs may leave vacant or partially vacant ecological niches that other species may inhabit. Along with adult trapping and “dipping” for larvae, presence and relative abundance of target and non-target species could be monitored through a molecular technique such as eDNA that detects trace DNA shed by mosquito larvae and pupae in water and sediment samples (Boerlijst et al., 2019; Krol et al., 2019). As previously mentioned, it is essential to rigorously monitor for drive-resistant alleles, as early detection is key to preventing their spatial spread, and the more time that passes, the more likely they are to emerge. For both replacement and suppression strategies, epidemiological data should continue to be monitored post-release, and may help to signal program failures.

Gene drive-modified mosquito projects are unique in the field of vector control in that they involve transgenes that could potentially spread on a wide scale. This means that, in certain cases, monitoring will be required on a wide scale. When a gene drive system is only intended to spread locally, for instance during a trial, it will be essential to monitor areas of unwanted spread for the intact drive allele, and when the gene drive system is intended to spread on a wide scale, it will be of interest to monitor on this scale for alternative alleles (e.g., homing-resistance alleles and non-functional effector genes). The scale, cost and projected effectiveness of wide-scale monitoring programs is important to consider as this is expected to be a major cost driver of gene drive mosquito projects as they advance from lab to field.

**FIGURE 1:**
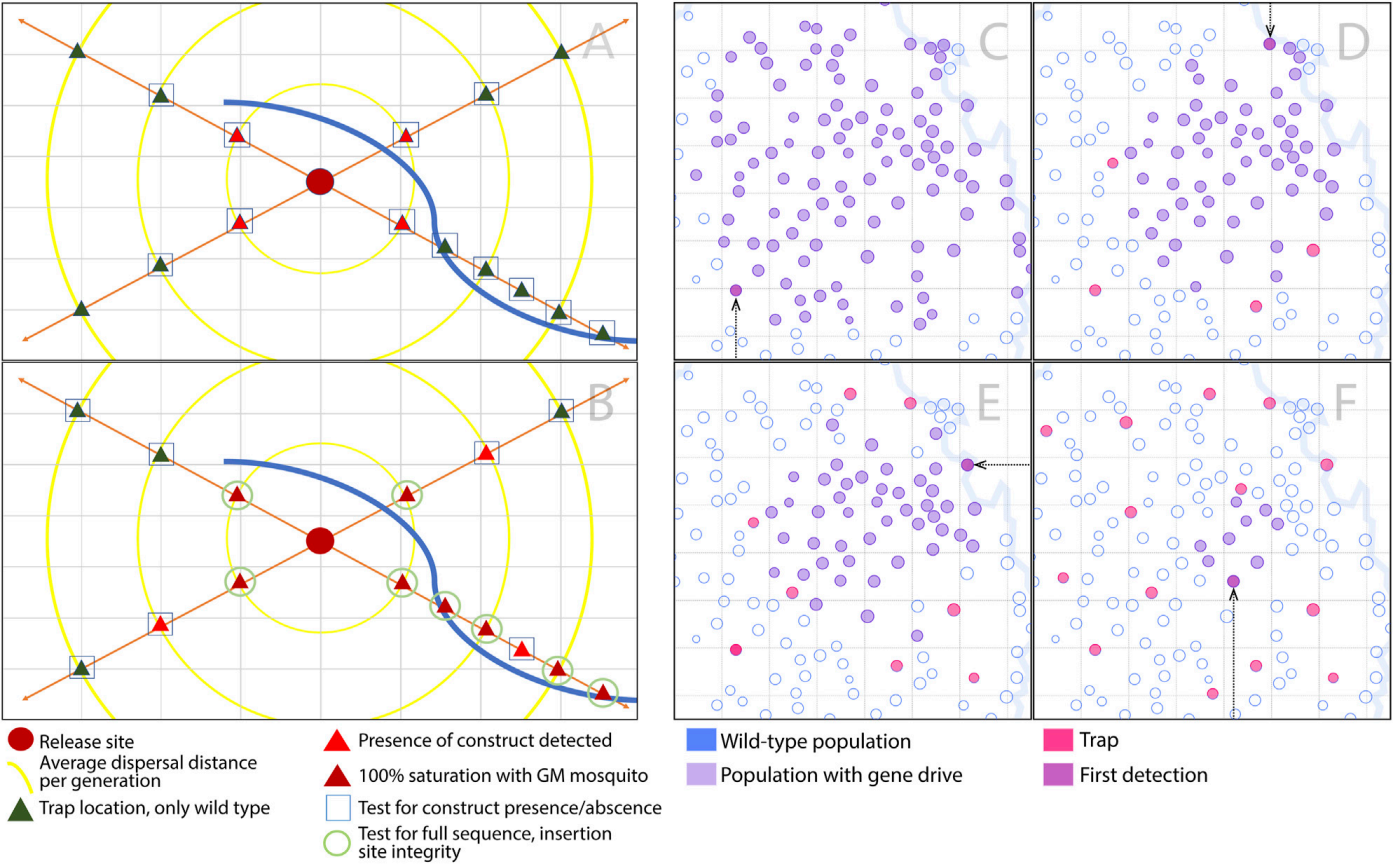
Optimal density and placement of traps to detect gene drive alleles, drive-resistant alleles and non-functional effector genes. **(A–B)** Transects may be used to optimize monitoring efforts to detect gene drive and alternate alleles in the vicinity of a release site during and post-release. **(A)** In the pictured scenario, during release, basic molecular tests would be conducted on adults to establish presence/absence of the construct and percent allele prevalence. Tested sites include primary sites (red triangles) adjacent to the release site (crimson circle), and secondary sites (green triangles) adjacent to primary sites, in order to capture early spread. In the pictured scenario, the lower-right arm of the sampling transect extends along a natural corridor of increased dispersal (e.g., prevailing wind direction). Traps along this section are placed at shorter intervals, extending further beyond the release site. **(B)** Post-release, molecular testing becomes less frequent, but more specific, once a site has reached high frequency or fixation for the gene drive construct. Once a large fraction of mosquitoes at a site has the construct, sampled mosquitoes having the construct will go on to have the construct and surrounding insertion site sequenced to verify construct integrity and functionality. Criteria should also be discussed regarding when, how and the frequency at which resistance in the malaria pathogen should be tested. **(C–F)** A mosquito metapopulation is denoted by a set of circles, each circle corresponding to a partially-isolated population connected to others by migration. Populations without the gene drive system are open circles with blue outlines, those with the gene drive are purple circles, those with traps are magenta circles, and the circle of first detection is plum, also denoted by an arrow. **(C)** In this simulation, with only 1 trap per 128 populations, the gene drive allele invades 101 populations before first detection. **(D–F)** As the number of traps is increased, the number of populations invaded at the time of detection declines: in this simulation, there are 56, 46 and 9 invaded populations for the cases of 5, 9 and 15 traps, respectively. Questions arise as to the density of traps required to detect a gene drive or alternate allele in time for it to be effectively managed, and how much investment would be required to achieve this.

Wide-scale monitoring programs to detect intact or alternative gene drive alleles stand to learn from experiences with invasive species, in which early invasions may be halted through effective monitoring programs (Koch et al., 2020). These programs often take into account the life history of the species in question, its predicted geographic distribution, expected pattern of spread, and the costs of monitoring activities. Multiple scenarios are then modeled to determine the most cost-effective option. A key result is that early detection is critical to minimizing the impact of an invasion and to preserving the possibility of local elimination (Holden et al., 2016). Future modeling analyses should explore the optimal density and placement of traps and frequency of sampling in order to detect gene drive alleles, drive-resistant alleles or non-functional effector genes while they can still be effectively managed (Figure 1).

Given the expected expense of these monitoring programs, cost-efficiency will be a priority. Difficult questions must be addressed regarding what can feasibly be achieved by wide-scale monitoring programs. Thus far, monitoring of the invasion of *An. stephensi* in the Horn of Africa (Seyfarth et al., 2019), and of *Ae. aegypti* in California (Lee et al., 2019), has resulted in documentation and genetic reconstruction of the invasions, rather than control or elimination. Hence, for *Anopheles* gene drive mosquito projects, how much investment is required to detect intact or alternate gene drive alleles in time for them to be effectively managed?

## Discussion

Low-threshold gene drive systems, whether intended for population replacement or suppression, pose significant demands on monitoring programs, both in terms of their persistence and potential to spread. Fortunately, lessons can be learned from examples of monitoring programs for genetic and biological control systems in *Ae. aegypti*, including analogs of high-threshold replacement *via Wolbachia*, and suppression via RIDL. Many of the monitoring priorities before, during and after a release also hold for low-threshold gene drive systems, especially at the stage of confined field trials. However, it should be noted that these case study systems have all been engineered in *Ae. aegypti*, while the low-threshold systems that are furthest along in development are all in *Anopheles* species. Hence, while many of the high-level considerations apply, consideration must also be given to their biological differences - greater dispersal distances, separation of breeding and blood-feeding sites, and trapping methods for *Anopheles* species. In parallel, methodology used for planning monitoring programs for invasive species appears to apply well to the detection of intact or alternative gene drive alleles; however, open questions remain as to what can be achieved with available resources. This will be an interesting area of research as this potentially transformative technology advances from lab to field.

## Data Availability

The original contributions presented in the study are included in the article, further inquiries can be directed to the corresponding author.
